# Pasteur’s Experiments Concerning Contagious Diseases

**Published:** 1883-03

**Authors:** 


PASTEUR'S EXPERIMENTS CONCERNING CONTAGIOUS DISEASES.—Our readers are acquainted with the experiments of Professor Pasteur, wherein the microbe or bacterium of splenic fever and the organisms peculiar to typhoid fever were successfully "cultivated" in the laboratory of the great French investigator.

All his aim is to find what microbe, germ, bacterian or organism lies at the basis of contagious and epidemic diseases, in order that by *inoculation* he may save human and valuable animal life.

So assured is the French Government of the utility and practicability of his aim that 50,000 francs have been granted him to pursue his researches.

Thus far Pasteur has found that microbes of contagious diseases are all subject to the same law, and that exposure *to free oxygen* during their period of cultivation reduces their virulence. All concede the value of this discovery. Contending factions and rival nationalities—in the medical world at least—are for once in accord, when they recognize how great an advance in science and public hygiene has been made by the steady work of one man.

Hydrophobia is now the subject of discussion and investigation. Pasteur took from the mouth of a hospital patient—who died of hydrophobia—the thick, viscid, frothy saliva and inoculated a healthy rabbit with some of it. The animal died in three days, and on microscopic examination of its blood, was seen a new microbe.

Now when, at different times, he repeated his inoculation, it was found that all the rabbits did not have hydrophobia, but some died of septicæmia.

But logic has more than one method ; *a priori* and *a fortiori* are different, yet by each can we prove and by each disprove.

Pasteur examined the saliva of individuals dying of other diseases and in it was the "new microbe." This was discouraging, for hydrophobia at least ; but accepting this complete set-back philosophically, he cultivated some seventy or eighty generations, and the last descendants were as potent to kill when inoculated into rabbits as the first, *i.e.*, that in the saliva taken directly from the mouth of one dying of hydrophobia.

Now it was to be noted through every step of cultivation, that all access of air was prevented. But when cultivation took place in the presence of free oxygen, the fluids or organisms very rapidly lost all power of producing disease in rabbits when inoculated.

He was able, however, to prolong the life of the organisms for a period varying from two to seven weeks ; and succeeded in obtaining a cultivated form which was capable of producing the same relationship to the acutely fatal disease as the disturbances following a vaccination do to small-pox.

He found, moreover, that hydrophobia could not be "given" to those Guinea-pigs or rabbits who had suffered the mild attack induced by inoculation of the organism that had been cultivated in the presence of free oxygen.

Hence Pasteur has succeeded. He has proved that the hydrophobia poison lurks in the saliva ; that it can be cultivated ; and that when cultivated in a certain way, it can be used as "vaccine," the animal vaccinated therewith being thenceforward safe from hydrophobia.

How much farther these experiments can be carried, and how many diseases they will deal with seems only to be decided by the time and money at the command of the investigator. The latter requisite the Government seems willing to supply ; we wish that time were also at its disposal.

